# Assessment of Physicochemical Properties, Cytotoxicity, Antimicrobial Activity, and Flexural Strength of Self-Cured Acrylic Resin With Silver Nanoparticles on Delaminated Clay for Removable Appliances

**DOI:** 10.1155/ijod/7939455

**Published:** 2025-04-07

**Authors:** Reza Mahmoudi Anzabi, Baharak Divband, Mustafa S. Tukmachi, Samin Vahedifar, Mahdiyeh Esmaeilzadeh, Fatemeh Yeganeh Sefidan, Morteza Jahanbani, Ali Rafighi

**Affiliations:** ^1^Department of Orthodontics, Faculty of Dentistry, Tabriz University of Medical Sciences, Tabriz, Iran; ^2^Dental and Periodontal Research Center, Tabriz University of Medical Sciences, Tabriz, Iran; ^3^Department of Inorganic Chemistry, Faculty of Chemistry, University of Tabriz, Tabriz, Iran; ^4^Department of Prosthodontics, College of Dentistry, University of Baghdad, Baghdad, Iraq; ^5^Student Research Committee, Faculty of Dentistry, Tabriz University of Medical Sciences, Tabriz, Iran; ^6^Department of Bacteriology and Virology, Faculty of Medicine, Tabriz University of Medical Sciences, Tabriz, Iran; ^7^Department of Orthodontics, Faculty of Dentistry, Babol University of Medical Sciences, Babol, Iran

**Keywords:** antibacterial, biocompatibility, clay, cytotoxicity, orthodontic appliances, removable, silver nanoparticle

## Abstract

**Background and Aim**: Silver nanoparticles represent a widely utilized nanotechnology product, prized for their versatile properties including electrical and thermal conductivity, and antimicrobial efficacy. This study aimed to assess the impact of incorporating silver nanoparticles-delaminated clay nanohybrid (AgNPs-DC) into acrylic resin on its antibacterial and antifungal characteristics, flexural strength, alongside evaluating the toxicity, and biocompatibility of the resultant composite.

**Materials and Methods:** Two concentrations of silver nanoparticles were initially integrated into the clay. Subsequently, cold-cure poly-methyl methacrylate (PMMA) was modified with varying percentages (0.2, 0.5, 1, and 1.5 wt%) of AgNPs-DC. Various analytical techniques including X-ray diffraction (XRD), field emission scanning electron microscopy (FE-SEM), energy-dispersive X-ray (EDX) analysis, flexural strength, and elemental mapping were utilized to verify synthesis and determine physiochemical properties. Cytotoxicity assessments were conducted using mouse fibroblast cell line (L929), while antibacterial activity against standard strains of *Escherichia coli* (*ATCC 25922*), *Enterococcus faecalis* (*ATCC 29212*), and *Candida albicans* (*ATCC 10239*) were evaluated using the colony count method.

**Results:** EDX analysis confirmed the presence of clay and silver nanoparticles in the final acrylic resin. Samples with higher concentrations of silver nanoparticles (10%), AgNPs-DC (1.0% and 1.5%), and synthesized acrylic powder (80 µg/mL) exhibited increased cytotoxicity, with diminished cell viability after 5 days of incubation. Moreover, an escalation in AgNPs-DC concentration correlated with a significant reduction in colony counts of *E. faecalis*, *E. coli*, *and C. albicans* in groups with 5wt% of silver nanoparticles (*p* =  0.018, *p* <  0.001, and *p* =  0.004, respectively). Encapsulation of AgNPs-DC within the polymer mitigated its toxicity, while higher concentrations of silver nanoparticles (10%) demonstrated enhanced antimicrobial properties. The mean flexural strength in groups with concentrations of 0.2 and 0.5 wt% of AgNPs-DC was significantly higher than in groups with concentrations of 1 and 1.5 wt% of AgNPs-DC. (*p* <  0.05).

**Conclusion:** In conclusion, this study underscores the potential of PMMA modification with AgNPs-DC nanoparticles to confer satisfactory antibacterial and antifungal characteristics. Nonetheless, the cytotoxicity of the synthesized polymer increased with nanoparticle concentration and duration. Notably, ball milling proved effective in reducing nanoparticle aggregation.

## 1. Introduction

Removable orthodontic appliances (ROAs) find widespread use in functional treatments, minor tooth adjustments, and postfixed orthodontic treatment retention [1]. Despite their myriad benefits, a significant concern looms: the accumulation of microbial biofilm on appliance surfaces, including wire components or acrylic base plates [1]. Polymethyl methacrylate (PMMA), renowned for its commendable mechanical properties, biocompatibility, availability, cost-effectiveness, and favorable esthetics, has become ubiquitous in the fabrication of complete and partial dentures, as well as ROAs [2, 3].

Conversely, the porosity of PMMA offers a conducive substrate for microbial growth and development [1, 4, 5]. Research underscores the superiority of heat-cure acrylics over self-cures, owing to their higher degree of conversion, resulting in reduced water absorption and porosity [1, 6, 7]. Additionally, the unpleasant odor emitted by some microorganisms inhabiting orthodontic base plates adversely affects patient compliance [6]. ROAs may also elevate the levels of *S. mutans and lactobacillus bacteria* in saliva and dental plaque, significantly heightening the risk of caries or gingival inflammation [7, 8]. Consequently, antimicrobial chemical interventions, such as chlorhexidine gluconate mouthwash, are recommended, as regular brushing alone may not effectively eradicate microorganisms. However, the gold standard substance falls short in many cases due to its associated side effects, including alterations in taste perception and tooth discoloration [7]. Given these limitations, the exploration of new materials with antimicrobial properties and acceptable physical characteristics for acrylic base plates holds promise.

Nanomaterials, with their favorable attributes, emerge as a viable option for augmenting the properties of acrylic base plates [9]. Extensive research has delved into the antimicrobial prowess of these particles and their application in orthodontic materials [9–11]. Silver nanoparticles stand out as one of the most widely employed nanotechnology products, owing to their advantageous properties, including malleability, electrical and thermal conductivity, and antimicrobial activity [12–15 ]. These nanoparticles exhibit exclusive antimicrobial effects against various microorganisms, such as *Streptococcus mutans*, *Staphylococcus aureus*, *Staphylococcus epidermidis*, *E. coli*, *and Candida albicans* [16–18]. However, concerns regarding nanoparticle toxicity necessitate innovative strategies for their safe incorporation into dental materials [19–21].

In response to this challenge, a novel technique for integrating metal oxide nanoparticles into polymer materials has been introduced, utilizing a stabilizer with larger dimensions and a porous matrix to mitigate accumulation, aggregation, and leaching from dental material compounds [22]. One such method involves stabilizing nanoparticles within the matrix network of inorganic aluminosilicates, such as zeolites [4] and clay minerals, known to enhance the antimicrobial properties of nanoparticles [23, 24]. Clay's layered structure entraps nanoparticles, facilitating the maintenance of their antimicrobial properties while preventing accumulation [25].

To our knowledge, no study has yet explored the effects of incorporating encapsulated silver nanoparticles (AgNPs) within the layered clay network (DC) into PMMA. The primary objective of this study is to assess the impact of adding AgNPs-DC to self-cure PMMA acrylic resin on its antibacterial properties against two bacterial strains (*Escherichia coli* (*ATCC 25922*), *Enterococcus faecalis* (*ATCC 29212*)), *and C. albicans* (*ATCC 10239*). Additionally, the study aims to evaluate the composite's flexural strength, toxicity, and biocompatibility offering valuable insights into its potential clinical utility.

## 2. Methods and Materials

In this experimental noninferiority trial, cold-cure acrylic resin (PMMA) was enhanced with silver nanoparticles-delaminated clay nanohybrid (AgNPs-DC). All materials utilized in this study were sourced from Sigma-Aldrich Co. The procedures undertaken were approved by the Ethics Committee of Tabriz University of Medical Sciences, with ethical approval number IR.TBZMED.VCR.REC.1400.223.

### 2.1. Synthesis of Silver Nanoparticles-Delaminated Clay Nano Hybrid (AgNPs-DC)

The synthesis of silver nanoparticles-delaminated clay nanohybrid (AgNPs-DC) commenced with the treatment of clay using an acid washing method. Initially, the clay was mixed with distilled water for 6 h, followed by smoothing and drying. Subsequently, a solution of hydrochloric acid with a concentration of 0.1 mol/L was prepared, and the clay was immersed in it for 24 h at room temperature, with stirring at a speed of 600 rpm to facilitate acid treatment. In the subsequent step, a solution of silver nitrate (AgNO3) with a concentration of 1 mol/L was added to the suspension of delaminated clay (DC) in water to achieve 5 wt% (Ag1-DC) nanohybrid. The mixture underwent stirring for 12 h at room temperature to ensure effective ion exchange between the silver nitrate and the clay matrix. In the next step, for the overall synthesis containing a higher percentage of silver, a ratio of 90 : 10 (weight/weight percentage) of silver chloride to overall was chosen so that the amount of silver is about 10% of the overall weight. In this way, aggregate particles containing silver nanoparticles (Ag2-DC) were synthesized. Following ion exchange, the synthesized nanocomposites were subjected to centrifugation, washing, and subsequent drying at room temperature to obtain the final AgNPs-DC nanohybrid.

### 2.2. Specimen Preparation and Structural Analysis

In this investigation, SR Triplex Cold orthodontic self-curing acrylic resin, manufactured by Ivoclar Vivadent AG/Liechtenstein, was utilized. The synthesized AgNPs-DC nanohybrid powder was incorporated into the Triplex Cold self-curing polymer acrylic powder at concentrations of 0.2, 0.5, 1, and 1.5 wt%. Following manual blending, the composite powder underwent a 20-min mixing process using a ball milling machine to ensure thorough homogenization. Subsequently, eight experimental groups and a control group were established (see Table 1).

#### 2.2.1. X-Ray Diffraction (XRD)

XRD analysis was employed to examine the crystalline structure of the acrylic resin powder samples integrated with the synthesized nanoparticles. This analysis was conducted utilizing the Bruker D8 ADVANCE XRD system (Germany), with the X-ray generator operated at 40 kV and 30 mA. The reflected radiations from the samples were recorded at room temperature within the angular range of 2*θ* = 4–70°, and a graph depicting their reflection intensity was generated. The presence or absence of nanocomposite formation was determined based on the peak height and position.

#### 2.2.2. Field Emission Scanning Electron Microscopy (FE-SEM), Energy Dispersive X-Ray (EDX) Analysis, and Elemental Mapping

Field emission scanning electron microscopy (FE-SEM) coupled with energy-dispersive X-ray (EDX) analysis was utilized for morphological and microstructural examination of the acrylic resin containing synthesized nanoparticles. X-ray scattering spectroscopy facilitated structural and elemental analysis of the samples. Prior to scanning, the surface of the nanocomposites underwent gold coating to ensure conductivity. SEM was conducted at room temperature with 20 kV and 6 μm magnification. Furthermore, elemental mapping analysis was conducted utilizing wavelength-dispersive X-ray spectroscopy (WDS) to assess the uniform distribution of nanoparticles and elements within the polymer samples.

#### 2.2.3. Atomic Absorption Spectroscopy

The concentration of silver nanoparticles was determined through electro-thermal absorption spectroscopy and MS-ICP analysis. Calibration of the instrument for nanoparticle quantification involved the use of a standard solution and the construction of a standard curve diagram. The standard solution of metal ions was prepared through dilution of standard solutions, and measurements were repeated thrice for accuracy.

### 2.3. Cytotoxicity Assessment via MTT Assay

The MTT assay, a widely employed colorimetric method, evaluates cell viability through the reduction of yellow tetrazolium crystals, namely 3-[4,5-dimethylthiazol-2-yl]-2,5-diphenyl-tetrazolium bromide (MTT), catalyzed by succinate dehydrogenase enzyme to form blue insoluble formazan crystals. This assay enables the assessment of various cell responses to external stimuli such as growth factors, cytotoxic agents, nanoparticles, radiation, and other chemical compounds with high repeatability, accuracy, and sensitivity.

In our study, all procedures, from cell culture initiation to result interpretation via ELISA, were meticulously executed in 96-well microplates. Cytotoxicity evaluation of the samples was evaluated by a cell viability assay using mouse fibroblast cell line (L929), according to the UNI EN ISO 10993/2009 [26]. The cultured cells were then dissociated with trypsin, irrigated, and counted. A density of 3 × 10^4^ cells were seeded in each well (containing 100 µL of cell culture medium supplemented with penicillin and streptomycin) of three different 96-well plates (six wells for each group). The 96-well plates were then separately incubated for 24, 72, and 120 h at 37 °C. The supernatant of each well was eliminated, and plates were incubated for another 2 h in a culture medium without FBS to remove the effects of growth factors. The supernatant of each well was again removed and replaced with FBS 1% culture medium containing different concentrations of synthesized acrylic powder (60 and 80 µg/mL). Six wells in each plate which contained conventional acrylic, were considered as positive control.

In the next step, 30 µL of MTT solution (5 mg/mL) in Phosphate-Buffered Saline (PBS) was added to each well. Plates were incubated for 4 h at 37 °C. Then, 180 µL of each well's supernatant were removed, and 160 µL of dimethyl sulfoxide and 20 *μ*L Sorenson buffer were added, and shaken for 20 min until the purple formazan crystals dissolved. Finally, the ELISA reader device (Immunoskan MS, BDSL, Pirbright, UK) was used to evaluate the absorbance wavelength at 570 nm. We used the following formula to calculate cell viability at different concentrations:

  $$\begin{array}{c} \%Viability\text{ (of sample)}=\left[\left(\text{A sample}-\text{A blank}\right)\right]/\left(\text{A control}-\text{A blank)]}\times 100\right) \end{array}$$

⁣^*∗*^A: Absorbance.

This meticulous methodology ensured precise evaluation of cytotoxic effects and provided valuable insights into the impact of synthesized nanoparticles on L929 cell viability.

### 2.4. Sample Preparation for the Antimicrobial Test

Samples were first rinsed with 70% ethanol and then irradiated with ultraviolet rays for 20 min to be sterilized. According to Table 1, the specimens were coated in a sterile 96-well plate with six repetitions. The acrylic without nanoparticles was placed in the first six wells and considered the positive control group. An acrylic sample containing silver nanoparticles with high concentration and proven antimicrobial properties [27] was also considered a negative control.

The colony count method was used to determine the antibacterial activity of samples against standard strains of *Escherichia coli* (*ATCC 25922*), *E. faecalis* (*ATCC 29212*), *and C. albicans* (*ATCC 10239*). The microbial standard strains were cultivated on brain-heart infusion (BHI) agar (Merck, Darmstadt, Germany) under sterile conditions for 24 h at 37 °C. After the incubation period, the standard 0.5 McFarland concentration of strains was prepared in sterile BHI broth, which is equivalent to 1.5 × 10^8^ colony forming unit per milliliter (CFU/mL). One hundred microliters of each microbial suspension were inoculated to 2 wells of each concentration of samples and incubated for 24 h at 37°C. Several dilutions (10^−1^, 10^−2^, 10^−3^, 10^−4^…) were prepared to count the number of microbial colonies in each well. Ten microliters of dilute microbial suspensions were inoculated on the surface of the BHI agar plate and then spread using the spread plate technique and incubated for 24 h at 37 °C. The plate with 30–300 cells was selected for counting, and the result was then reported in CFU/mL.

### 2.5. Flexural Strength Test

**Figure 1:**
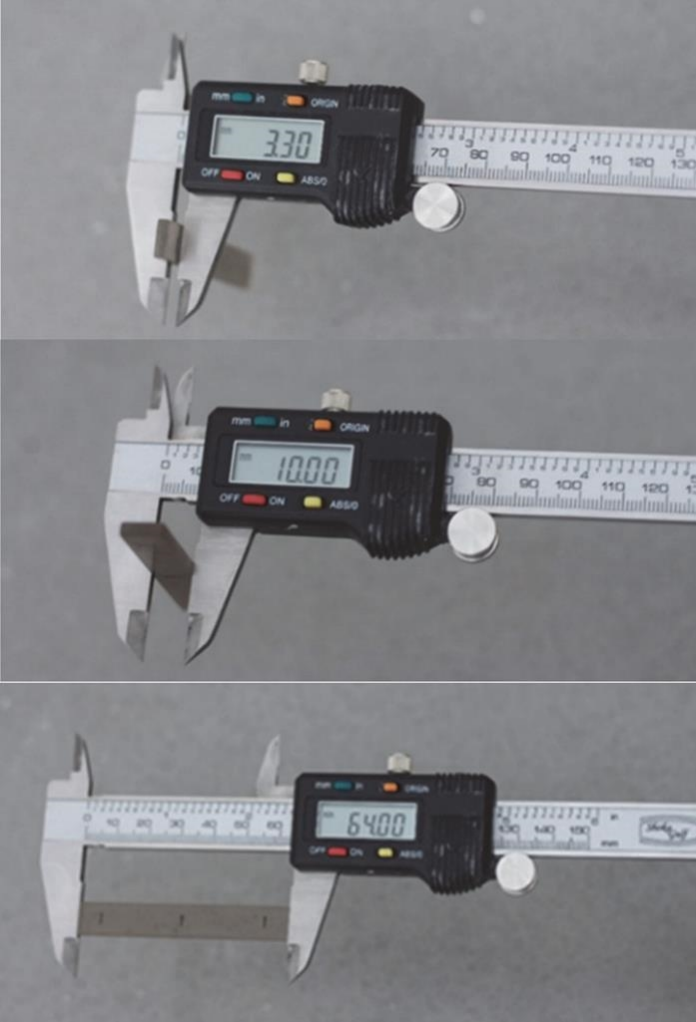
Confirming the dimensions of acrylic specimens to the dimensions recommended in the ISO 20795-2:2013 standard using a micrometer.

**Figure 2:**
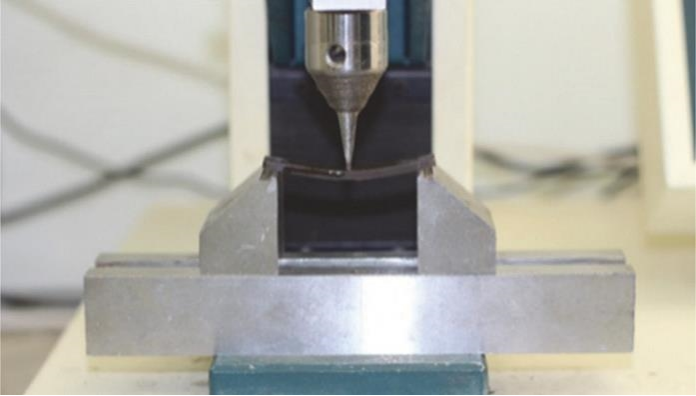
Three-point bending test using Universal testing machine (HOUNSFIELD-H5KS).

According to the ISO 20795-2:2013 guidelines [28], three-point bending test was performed to evaluate the flexural strength of acrylic specimens using Universal testing machine (HOUNSFIELD-H5KS). The loading plunger of machine was calibrated to provide a constant displacement rate of 5 mm/min and the distance between centers of the supports was set to 50 mm. Nine specimens (*n* = 9) from each group and a total of 45 specimen strips were prepared with a length of 64 mm, a width of 10 ± 0.2, and a height of 3.3 ± 0.2 mm according to the ISO 20795-2:2013 standard (Figure 1), and according to the instructions of ISO 20795-2:2013, all faces of the specimens were wet-grinded and smoothed with the metallographic grinding papers to the required width and height. Three measurements of the specimen height along the long axis were taken to an accuracy of ±0.01 mm using a micrometer. The specimens shall be flat and have an even height. The deviation between the three measurements along the long axis shall be no more than ±0.02 mm. Then the specimens were stored in the water at a temperature of 37°C for (50 ± 2) hours prior to flexural test. After the mentioned period, the specimens were taken out of the water storage one by one and immediately placed symmetrically on the supports of the flexural test rig (Figure 2). The force on the loading plunger was increased from zero, uniformly, using a constant displacement rate of 5 mm/min until the specimen breaks. The maximum load measured to break each sample, in newtons, was read from the device and the flexural strength (*σ*), in megapascals, was calculated using the following equation:

 

  $$\begin{array}{c} \sigma =3Fl/2bh^{2} \end{array}$$

Where

*F* is the maximum load, in newtons, exerted on the specimen; *l* is the distance, in millimeters, between the supports; *b* is the width, in millimeters, of the specimen measured immediately prior to water storage; *h* is the height, in millimeters, of the specimen measured immediately prior to water storage.

The results were reported as descriptive statistics in the form of average (standard deviation) or number (percentage). SPSS version 17 software was used for data analysis.

## 3. Results

### 3.1. XRD

**Figure 3:**
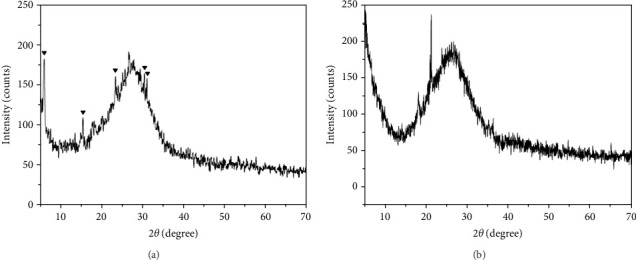
(A) the X-ray diffraction (XRD) patterns related to the acrylic resin containing D1, (B) the XRD patterns related to the acrylic resin containing D2.

Figures 3A and B show the XRD patterns related to the acrylic resin containing D1 and D2, respectively. The marked peaks in the figures correspond to the silver nanoparticles-delaminated clay nanohybrid (AgNPs-DC) structure. As can be seen, the height of peaks is elevated with the increase in the percentage of AgNPs.

### 3.2. FE-SEM, EDX, and MAP

**Figure 4:**
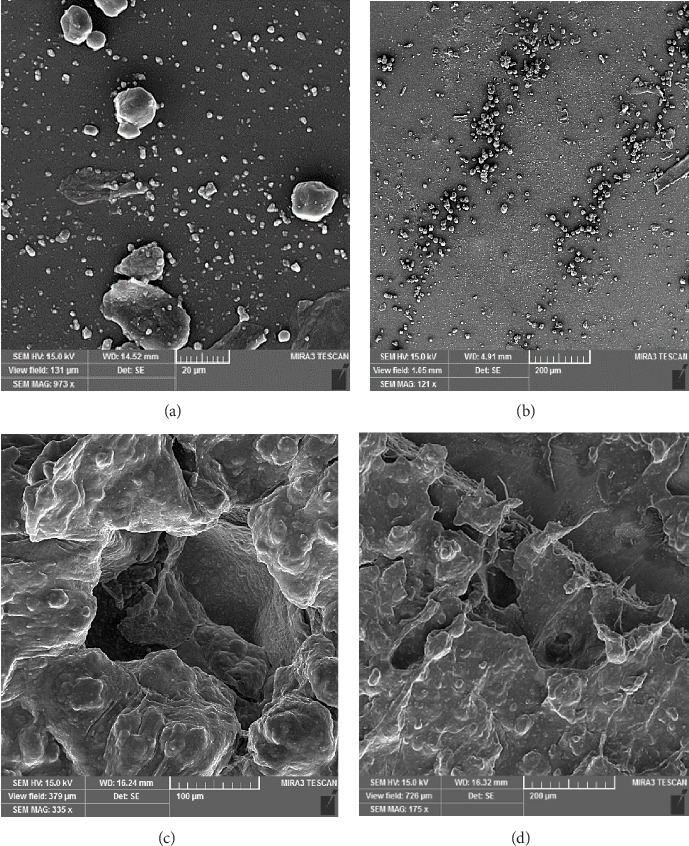
Field emission scanning electron microscopy (FE-SEM) images from the acrylic resin containing (A) 0.2% Ag1-DC, (B) 0.5% Ag1-DC, (C) 1.0% Ag1-DC, (D) 1.5% Ag1-DC.

**Figure 5:**
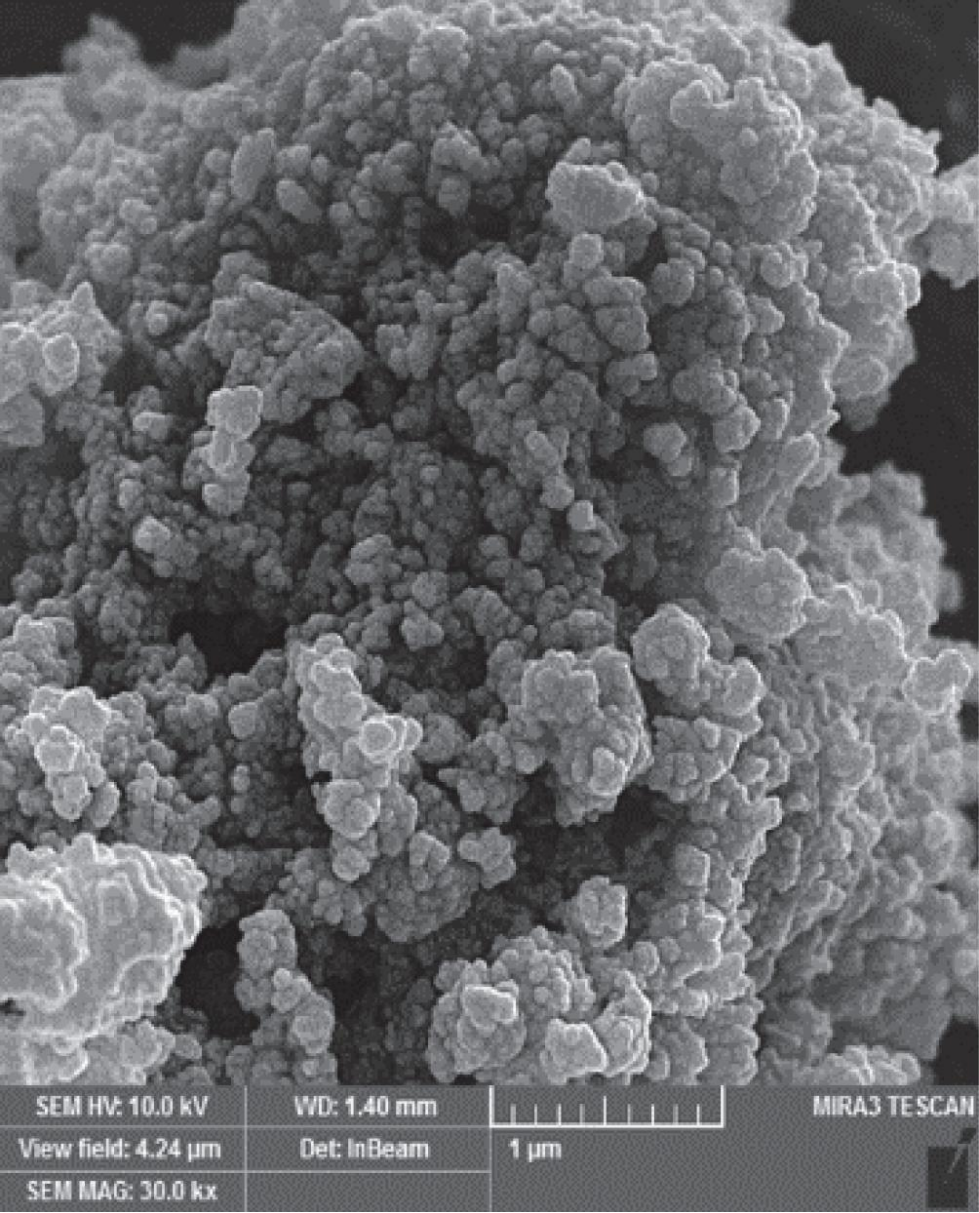
Field emission scanning electron microscopy (FE-SEM) images from the Ag2-DC nanocomposite.

**Figure 6:**
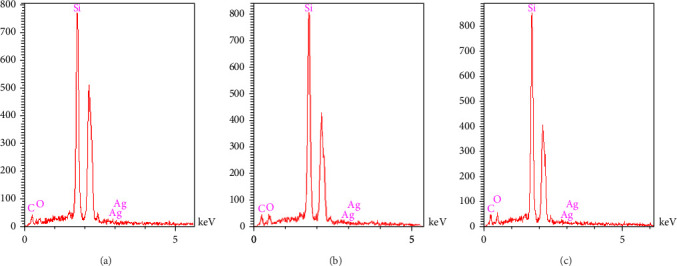
Energy-dispersive X-ray (EDX) analysis related to the acrylic resin containing (A) 0.5 wt% Ag1-DC, (B) 1 wt% Ag1-DC, (C) 1.5 wt% Ag1-DC.

**Figure 7:**
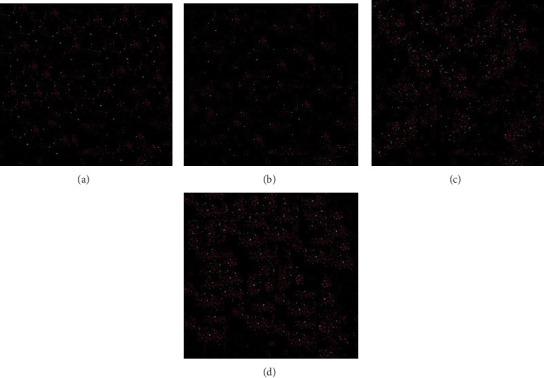
MAP analysis related to the (A) 0.2 wt% Ag2-DC, (B) 0.5 wt% Ag2-DC, (C) 1 wt% Ag2-DC, and (D) 1.5 wt% Ag2-DC (red and green dots, respectively, indicate Si and Ag atoms).

Analysis FE-SEM images from the acrylic resin containing different concentrations of Ag-DC (A1–D1) are shown in Figure 4. There were some raptures on the surface of the polymer as a result of electron beam irradiation. The disintegration of the polymer was more evident in group A, where the concentration of nanoparticles was 0.2%. The accumulation of nanoparticles and the polymer disintegration was also explicit in group B. The resistance against deterioration has enhanced with the increase in the concentration of nanoparticles, which can be seen in groups C (1%) and D (1.5%). Since the amount of AgNPs did not significantly affect the resultant image, FE-SEM scans from Ag2-DC samples (higher concentration of AgNPs) were prepared just before mixing with acrylic resin (Figure 5). The higher concentration of nanoparticles has made the clay layers stick together. The results of total elemental and structural analysis (EDX) analysis of polymerized acrylic resin containing Ag/DC nanoparticles with concentrations of 0.5, 1, and 1.5 wt% showed the presence of silicon, silver, and oxygen atoms, confirming the presence of clay and silver in acrylic resin (Figure 6). Considering that there was no trace of polymer left in sample A (0.2 wt%), EDX analysis was meaningless and was not performed in group A. Moreover, in the EDX-mapping images, the elements in all samples showed an almost uniform and homogeneous distribution, and in some areas, accumulation was seen (Figure 7), which was compatible with the scanning electron microscope images. As can be seen, the number of red and green dots in the images increases with the increase in the percentage of nanoparticles in acrylic. Due to the fact that there was no trace of polymer left in sample A, the mapping analysis showed an extremely large accumulation of nanoparticles.

### 3.3. Atomic Absorption Spectroscopy

The atomic absorption spectroscopy results were in good agreement with the EDX images of the samples. The total percentage of silver, aluminum, and silicon in samples A1, B1, C1, and D1 was 0.18, 0.49, 0.96, and 1.54, respectively. The sum percentage of silver, aluminum, and silicon in groups A2, B2, C2, and D2 was also 0.21, 0.52, 1.06, and 1.49, respectively. These percentages show an acceptable match with the percentages of Ag/DC nanohybrid mixed with acrylic resin which were 0.2, 0.5, 1, and 1.5 wt%, respectively.

### 3.4. The MTT Cytotoxicity Assay

Two concentrations of modified acrylic powder (60 and 80 µg/mL) were prepared for cytotoxicity assessment. The experimental groups included the control, polymer-containing groups (A1, B1, C1, D1, A2, B2, C2, D2), and groups without polymer (AgNPs-DC (CI) and AgNPs (CII)). The control group exhibited 100% survival rate.

**Figure 8:**
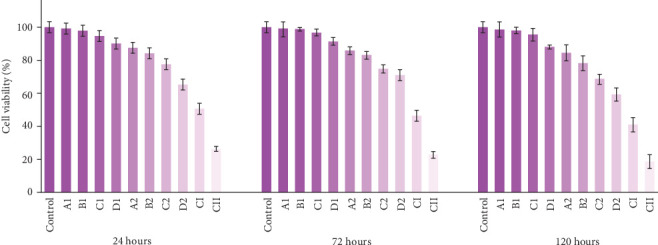
Cell viability for concentration of 60 µg/mL after 1, 3, and 5 days.

**Figure 9:**
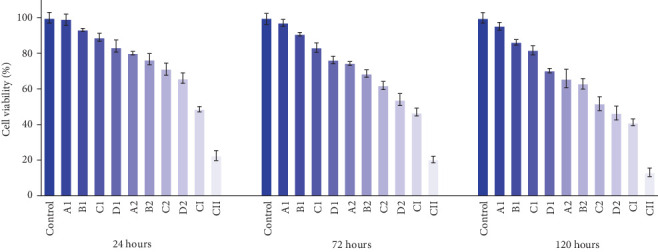
Cell viability for concentration of 80 µg/mL after 1, 3, and 5 days.

The results indicated a dose-dependent cytotoxic effect, with increased silver concentrations in the nanocomposite and acrylic resin correlating with reduced cell viability. Additionally, incubation time (24, 72, and 120 h) significantly influenced cytotoxicity, with prolonged exposure leading to lower survival rates. As expected, the higher concentration (80 µg/mL) resulted in greater cytotoxicity than the 60 µg/mL group (Figures 8 and 9).

Interestingly, the negative control groups (CI and CII), containing 1.2 µg/mL of AgNPs-DC and AgNPs without polymer, respectively, exhibited the highest cytotoxicity among all study groups. However, as shown in the figures, encapsulation of AgNPs in the clay matrix, as well as the incorporation of the AgNPs-DC nanocomposite within the polymer matrix, significantly reduced cytotoxicity, suggesting a protective effect of these formulations.

### 3.5. The Antimicrobial Properties

The antibacterial characteristics were most pronounced in acrylic resin formulations with higher proportions of nanoparticles (Groups A2 to D2). Additionally, the antimicrobial efficacy increased with higher concentrations of silver nanoparticles within the nanocomposite (Groups D1 and D2 exhibited superior antimicrobial properties compared to Groups A1 and A2, respectively) (refer to Table 2). A notable discrepancy in colony counts of *E. faecalis*, *E. coli*, *and C. albicans* was evident among groups featuring five weight percent of silver nanoparticles (*p* = 0.018, *p* < 0.001, and *p* = 0.004, respectively).

### 3.6. Flexural Strength

**Figure 10:**
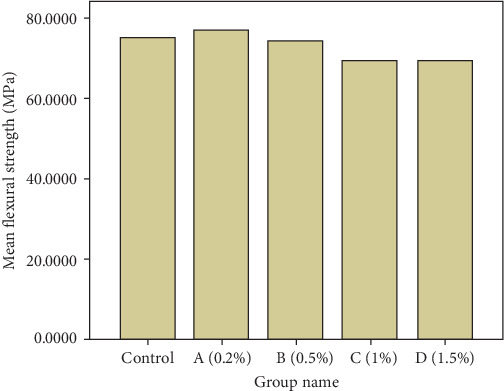
Boxplot diagram of the flexural strength of the studied groups (MPa).

**Figure 11:**
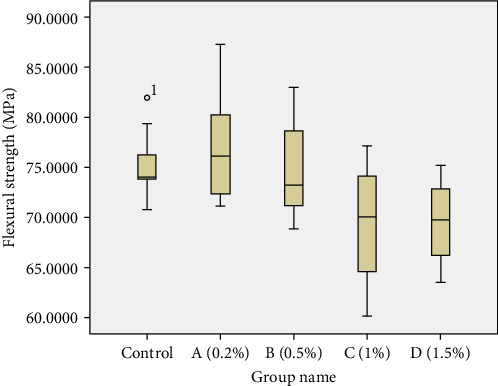
Bar graph of the mean flexural strength of the studied groups (MPa).

The results of the flexural strength are given in Figures 10 and 11. According to the Shapiro–Wilk test results, the data have a normal distribution (sig > 0.05). One-way ANOVA test showed that there was a significant difference between the studied groups (df = 4, *F* = 4.682, *p* = 0.003). The results of the flexural strength test showed that among the studied groups, the highest mean flexural strength of acrylic specimens belonged to group A (76.95 MPa), and the second highest mean flexural strength belonged to the control group (75.15 MPa). According to the results of the LSD test, there was no significant difference in the flexural strength between groups A, B, and the control group (*p* >  0.05). The lowest mean flexural strength corresponded to groups C (69.42 MPa) and D (69.49 MPa). There was no significant difference in mean flexural strength between groups C and D (*p* = 0.973). In general, the mean flexural strength in groups with concentrations of 0.2 and 0.5 wt% of Ag/DC was significantly higher than groups with concentrations of 1 and 1.5 wt% of Ag/DC (*p* <  0.05). It is noteworthy that despite the significant decrease in the mean flexural strength in groups C and D, all values were greater than 50 MPa, which is acceptable according to the ISO 20795-2 (2013) standard.

## 4. Discussion

This research aimed to assess the impact of incorporating varying concentrations of AgNPs-DC into cold-cure acrylic resin on its antibacterial efficacy postpolymerization. Cold-cure PMMA constitutes a prevalent material in the fabrication of ROAs. Following polymerization, residual monomer evaporation results in minor porosities within the polymer's framework. The oral cavity's microbial flora encompasses diverse microorganisms capable of colonizing various surfaces. Despite meticulous finishing and polishing, the acrylic base plate of ROAs provides a conducive environment for microbial growth. Microbial accumulation on acrylic resin amplifies the risk of dental caries and oral ailments, jeopardizing orthodontic treatment efficacy.

Volatile sulfide compounds (VSCs), including hydrogen sulfide and methyl mercaptan, are metabolic by-products of proteolytic, anaerobic, and gram-negative bacteria activities [27]. Given that functional ROAs necessitate prolonged wear, particularly among young patients, treatment outcomes heavily rely on patient cooperation. The malodor ensuing after several months emerges as a chief deterrent to patient compliance and motivation [1, 7, 8]. Previous investigations on prosthetic dentures, often composed of heat-cure PMMA, identify *Klebsiella pneumoniae* and *E. coli* as predominant colonizing microorganisms on acrylic bases [29]. The proliferation of gram-negative bacteria, notably within the *Enterobacteriaceae* family [30], is cited as a primary cause of malodor. Prior studies have postulated that incorporating antibacterial agents into acrylic resin may impede biofilm formation. Notably, the antibacterial properties of nanoparticles, particularly AgNPs, have garnered significant attention among dental material researchers. Clinical orthodontic applications have corroborated the antimicrobial efficacy of silver nanoparticles [16–18]. Farhadian et al. [8] demonstrated the inhibitory effects of AgNPs incorporation into acrylic orthodontic retainers on *Streptococcus mutans* colonization, aligning with the findings of this study. Biocompatibility and nontoxicity are pivotal considerations in nanomaterial utilization. Concerns regarding the cytotoxic effects of silver nanoparticles have prompted exploration into mitigating strategies such as employing larger stabilizers and porous matrices to curtail nanoparticle accumulation, aggregation, and leaching [19–22]. Various synthesis techniques have been proposed, including stabilizing nanoparticles within inorganic aluminosilicate matrices like clay minerals. Silver nanoparticles intercalated between clay layers' exhibit reduced release rates and subsequent toxicity [24]. The addition of nanoparticles at higher concentrations to acrylic resin is constrained by its adverse impact on mechanical properties. Thus, weight percentages ranging from 0.2% to 1.5% were investigated for their antimicrobial efficacy, alongside varying silver concentrations within the clay. Clay particles are commonly cited as biocompatible materials. Choi et al. [31] confirmed the safety of clay nanoparticles for biological applications, a finding corroborated by the present study, which observed no significant cytotoxicity across nanoparticle concentrations. Incorporating nanoparticles into cold-cure acrylic resin to enhance its antimicrobial properties can influence its mechanical attributes, notably flexural strength, a critical parameter outlined by the International Standard Organization (ISO 20795-2) [32]. Previous research by Esmaeilzadeh et al. [22]. evaluated the effects of ZnO/TiO/4A Zeolite addition to self-cure PMMA acrylic resin on flexural strength, indicating a significant reduction at high concentrations of nanoparticles, albeit remaining above the ISO standard threshold. Uniform dispersion and distribution of nanoparticles within the resin matrix are pivotal for optimizing physical and antimicrobial properties. Agglomeration risks escalate with increasing nanoparticle concentrations. Various methods, such as amalgamator usage [3, 22, 33] or ball milling, have been proposed to deal with undesirable accumulation. Ghaffari et al. [34] and Shirkavand et al. [3] used an amalgamator, which improperly dispersed the nanoparticles. Therefore, the ball milling method was used in the present study, which led to better dispersion and distribution.

This study employed FE-SEM and MAP analyses to scrutinize composite microstructure and homogeneity. To address aggregation challenges, ball milling was adopted over amalgamator usage, resulting in improved nanoparticle dispersion and distribution.

## 5. Conclusion

In summary, findings from this study demonstrate that incorporating AgNPs-DC nanohybrid into PMMA results in favorable antibacterial and antifungal characteristics. The toxicity of the produced polymer escalated with higher concentrations and duration of nanoparticle exposure. Additionally, ball milling proved to be an effective method for diminishing nanohybrid aggregation.

## Figures and Tables

**Table 1:** Test and control groups based on the percentage of nanoparticles added to PMMA powder.

Group	Nanoparticle type	Concentration
A1	Ag1-DC	0.2 wt%
B1	Ag1-DC	0.5 wt%
C1	Ag1-DC	1.0 wt%
D1	Ag1-DC	1.5 wt%
A2	Ag2-DC	0.2 wt%
B2	Ag2-DC	0.5 wt%
C2	Ag2-DC	1.0 wt%
D2	Ag2-DC	1.5 wt%
Control	None	0.0 wt%

Abbreviation: PMMA, poly-methyl methacrylate.

**Table 2:** The antimicrobial properties of different concentrations of synthesized acrylic resin.

Microorganism	Primary concentration (CFU/mL)	Concentration after 24 h (CFU/mL)								
		Control	A1	B1	C1	D1	A2	B2	C2	D2
*Enterococcus faecalis*	2.1 × 10^10^	Uncountable	1.2 × 10^7^	2.0 × 10^5^	8.4 × 10^4^	2.3 × 10^2^	3.1 × 10^5^	9.7 × 10^3^	2.3 × 10^3^	2.1 × 10^1^
			8.1 × 10^7^	4.2 × 10^5^	5.6 × 10^4^	2.5 × 10^2^	8.0 × 10^5^	41 × 10^4^	7.3 × 10^2^	4.0 × 10^1^
			1.2 × 10^7^	8.1 × 10^4^	8.0 × 10^3^	3.0 × 10^1^	6.2 × 10^5^	3.1 × 10^3^	5.6 × 10^2^	0
			1.1 × 10^8^	8.3 × 10^5^	4.3 × 10^3^	6.1 × 10^1^	9.7 × 10^4^	1.1 × 10^3^	4.1 × 10^2^	0
			6.1 × 10^6^	1.4 × 10^4^	2.3 × 10^3^	4.3 × 10^2^	6.8 × 10^4^	1.3 × 10^4^	2.1 × 10^2^	0
			5.1 × 10^7^	9.1 × 10^4^	6.0 × 10^4^	1.6 × 10^2^	1.1 × 10^5^	6.1 × 10^3^	6.2 × 10^2^	0

*Escherichia coli*	5.2 × 10^11^	Uncountable	1.4 × 10^5^	3.0 × 10^3^	5.1 × 10^2^	0	1.2 × 10^3^	2.6 × 10^1^	0	0
			1.6 × 10^4^	1.6 × 10^2^	3.3 × 10^2^	0	1.4 × 10^2^	3.5 × 10^2^	0	0
			2.2 × 10^4^	2.4 × 10^2^	1.6 × 10^1^	0	3.2 × 10^3^	1.4 × 10^1^	0	0
			8.7 × 10^4^	1.2 × 10^3^	2.7 × 10^1^	0	4.2 × 10^3^	3.5 × 10^1^	0	0
			8.4 × 10^5^	8.8 × 10^2^	2.3 × 10^2^	0	4.4 × 10^2^	5.6 × 10^2^	0	0
			4.3 × 10^5^	7.2 × 10^3^	4.0 × 10^1^	0	8.2 × 10^2^	8.4 × 10^1^	0	0

*Candida albicans*	4.3 × 10^10^	Uncountable	3.0 × 10^3^	6.0 × 10^3^	1.0 × 10^3^	6.0 × 10^1^	1.1 × 10^3^	5.4 × 10^2^	0	0
			7.2 × 10^4^	4.3 × 10^3^	1.6 × 10^2^	7.2 × 10^1^	4.3 × 10^2^	1.3 × 10^2^	9.1 × 10^1^	0
			1.1 × 10^4^	3.1 × 10^4^	3.1 × 10^3^	2.3 × 10^1^	1.9 × 10^1^	1.1 × 10^1^	0	0
			1.2 × 10^4^	1.5 × 10^3^	8.2 × 10^3^	4.2 × 10^2^	1.8 × 10^3^	2.3 × 10^1^	3.2 × 10^2^	0
			4.1 × 10^4^	1.3 × 10^3^	4.7 × 10^2^	8.7 × 10^1^	4.6 × 10^2^	4.3 × 10^2^	1.1 × 10^1^	0
			8.2 × 10^3^	3.2 × 10^3^	4.8 × 10^2^	1.2 × 10^1^	7.8 × 10^1^	6.3 × 10^1^	0	0

## Data Availability

The data that support the findings of this study are available on request from the corresponding authors.
